# Socio-cultural challenges and mental health needs of female sex workers in Bangladesh: a call for culturally sensitive interventions

**DOI:** 10.1186/s12939-025-02544-w

**Published:** 2025-10-06

**Authors:** Md. Ashiquir Rahaman, Farah Tabassum Shamma

**Affiliations:** Department of Clinical Psychology, University of Dhaka, Dhaka, Bangladesh

**Keywords:** FSW, Mental health, Stigma and discrimination, Culturally sensitive interventions

## Abstract

Female sex workers (FSW) in Bangladesh face multifaceted socio-cultural, legal, and economic challenges that significantly impact their mental health and well-being. With an estimated population of 200,000, FSW enter the profession primarily due to poverty, trafficking, forced marriage, and gender-based violence. The legal status of sex work remains ambiguous, exposing FSW to exploitation, violence, and limited access to justice. Stigma rooted in religious and cultural beliefs leads to exclusion from community and religious life, while children of FSW face social rejection and educational barriers. Studies report high prevalence of depression, anxiety, PTSD, and substance use disorders among Bangladeshi FSW, yet access to mental health services remains limited due to provider bias, legal obstacles, and lack of tailored interventions. Systemic barriers also restrict access to healthcare, banking, welfare, and vocational training. Addressing these issues requires culturally sensitive, community-based mental health interventions, including peer-led support, trauma-informed care, and sensitization of service providers. Policy reforms to decriminalize sex work and promote human rights are essential for sustainable change. Future research should explore community-driven strategies, the role of religious institutions in stigma reduction, and the specific needs of FSW and their children to inform effective, inclusive interventions and policy development.

Female sex workers (FSW) in Bangladesh face socio-cultural challenges that are deeply rooted in stigma and discrimination, unique to their profession. With an estimated population of 200,000, FSW operate in diverse settings, including streets, brothels, hotels, and residences, reflecting the fluid nature of their work [1]. In Bangladesh, FSW enter the profession through various pathways, primarily driven by systemic inequities. Economic necessity, affecting approximately 60% of FSW, is a leading cause, as poverty and lack of employment opportunities push women into sex work as a survival strategy [1]. Human trafficking is another significant factor, with an estimates from a Guardian article suggesting that 90% of FSW are trafficked, often sold by strangers, family members or husbands without their consent [2]. Forced marriages and gender-based violence also contribute, compelling women to escape abusive environments through sex work. The consequences of entering sex work as a profession are profound in Bangladesh’s socio-cultural context. They encounter legal complexities, as the legal framework surrounding sex work in Bangladesh is ambiguous. It is neither fully legalized nor explicitly prohibited, leaving FSW vulnerable to harassment and exploitation by law enforcement without legal protections [3]. Similarly, a report from the United Nations Development Programme on Asia and the Pacific notes that colonial-era legislation, such as the Suppression of Immoral Traffic Act, criminalizes aspects of sex work, creating a legal vacuum that restricts FSW’s access to justice [4]. Additionally, FSW face occupational hazards such as physical violence from clients, sexual assault, coercive control by pimps, and elevated risks of sexually transmitted infections (STIs), including HIV/AIDS, all of which heighten their marginalization [5]. Moreover, FSW face societal rejection, including exclusion from family and community networks, while their children endure stigma, such as bullying and school exclusion. This rejection, coupled with limited access to alternative livelihoods, traps many FSW in the industry, as societal attitudes and lack of reintegration support create insurmountable barriers to exiting. These challenges are further exacerbated by socio-economic disparities, including limited access to healthcare, education, and employment opportunities. The intersection of these factors significantly compromises the mental health and well-being of FSW. Addressing these issues requires immediate intervention, including the development of tailored mental health care services that acknowledge and respond to the unique needs and challenges of this marginalized population. Additionally, community awareness of the causes of sex work (e.g., trafficking as a form of coercion rather than choice) is essential for culturally sensitive interventions, as it fosters empathy and reduces victim-blaming.

FSW in Bangladesh face acute socio-cultural challenges rooted in the country’s Muslim-majority, collectivist culture. Religious stigma frames sex work as haram (forbidden), leading to exclusion from religious spaces, such as mosques refusing funeral rites for deceased FSW [6]. Community violence, exemplified by historical brothel evictions (e.g., the 2014 Tangail brothel closed after pressure from religious groups), reflects widespread rejection [7]. The children of FSW also face significant discrimination, including bullying and exclusion from schools due to their mothers’ profession, perpetuating cycles of marginalization. These challenges are amplified by cultural emphasis on family honor, which ostracizes FSW and their families. Street-based and hotel-based FSW face greater stigma than brothel-based FSW, creating a hierarchy of marginalization [1], which further exacerbates their mental health problems.

A systematic review across 26 low- and middle-income countries reports high prevalence of depression (41.8%), anxiety (21.0%), post-traumatic stress disorder (PTSD) (19.7%), and suicidal ideation (22.8%) among FSW [8]. In Southeast Asia, entrenched social and cultural factors, compounded by experiences of violence, further exacerbate their vulnerability to mental health disorders [9]. However, compared to other countries, data on Bangladeshi FSW are limited. A Chottogram-based Bangladeshi study highlighted alarming rates of mental disorders among FSW, including substance use disorder (22.0%), anxiety disorder (20.8%), and mood disorder (4.2%) [10]. Another study reported notably high rates of depression (62.0%), substance-induced psychotic disorder (18%), and antisocial personality disorder (8.0%) among FSW in Dhaka [11].

Despite these findings indicating a significant mental health burden, FSW encounter multifaceted barriers to accessing essential services, driven by systemic, structural, and cultural factors. In healthcare, 51% of FSW in Dhaka report barriers due to provider bias, judgmental attitudes, and high out-of-pocket costs [12]. Mental health services are particularly inaccessible due to a lack of FSW-specific programs and stigma from psychologists and psychiatrists [10]. Banking services are restricted, as FSW often lack identification documents and face prejudice from bank staff, limiting their financial independence. Educational and vocational training programs are inaccessible due to stigma and scheduling conflicts with work hours. Welfare benefits are largely unavailable, as FSW are excluded from government schemes due to their unregistered status. Problematic actors include healthcare providers (e.g., nurses refusing treatment), law enforcement (e.g., police extortion), NGOs (e.g., inconsistent funding), and community members who uphold myths portraaying FSW as immoral or disease vectors. These barriers are fueled by cultural beliefs rooted in religious prohibitions and public opinion that deems FSW undeserving of rights. These socio-cultural dynamics necessitate interventions that address both community attitudes and systemic exclusion.

To address the mental health needs of FSW in Bangladesh, culturally sensitive, community-based interventions tailored to the socio-cultural context are essential, alongside critical policy reforms. Peer-led support groups can provide safe spaces for counseling and peer support, fostering resilience and reducing isolation [13], while trauma-informed care delivered by trained providers can address experiences of violence and coercion [14]. Sensitization training for healthcare providers, police, and community leaders, emphasizing Islamic values of compassion (rahma) and justice (adl), can help reduce stigma. These interventions are feasible when aligned with Islamic ethical principles and supported by progressive religious leaders, as seen in Pakistan’s HIV programs [15]. Policy reforms are equally vital, including decriminalizing sex work by replacing punitive laws such as Sect. 290 of the Bangladesh Penal Code with policies that recognize sex work as legitimate labor, modeled on New Zealand’s framework [16], and enacting laws to protect FSW from violence and exploitation. Advocacy strategies should involve collaboration with FSW collectives like the Sex Workers Network Bangladesh, media campaigns to shift public opinion, and engagement with religious scholars to frame decriminalization as a human rights issue using Islamic ethical frameworks. Although resistance from conservative policymakers is anticipated, gradual reform through pilot programs and evidence-based advocacy offers a feasible path forward.

Moreover, comprehensive research is essential to inform targeted interventions. Existing studies show that FSW face healthcare barriers due to stigma and provider bias [12], with mental health services particularly inaccessible [10]. Legal obstacles, such as arrests under vague laws, further discourage FSW from seeking services [4]. Community culture, heavily influenced by religious norms, fosters opposition to sex work, as evidenced by brothel evictions [7], although some communities tolerate registered brothels while rejecting street-based FSW, creating a hierarchy of stigma [15]. Nevertheless, critical knowledge gaps persist, including a lack of longitudinal studies on the effectiveness of community-based interventions, insufficient research on the role of religious institutions in reducing stigma, limited data on the needs of FSWs’ children, and minimal exploration of intersectional factors such as ethnicity and rural versus urban settings. Future research should prioritize mixed-methods studies to evaluate peer-led and community driven interventions.

In doing so, we not only uplift the lives of FSW but also enhance the collective well-being of our communities by paving the way for a more inclusive and equitable society.

## Data Availability

No datasets were generated or analysed during the current study.
